# An unusual case of bone regeneration of a necrotic mandible with pathologic fracture in an elderly hemodialysis patient with medication-related osteonecrosis of the jaw: a case report and review of the literature

**DOI:** 10.1186/s13256-021-03206-5

**Published:** 2021-12-23

**Authors:** Kunio Yoshizawa, Akinori Moroi, Ran Iguchi, Akihiro Takayama, Junko Goto, Yutaka Takayama, Koichiro Ueki

**Affiliations:** 1 Division of Medicine, Department of Oral and Maxillofacial Surgery, Interdisciplinary Graduate School, University of Yamanashi, 1110 Shimokato, Chuo, Yamanashi 420-3898 Japan; 2 Department of Emergency and Critical Care Medicine, School of Medicine, University of Yamanashi, Yamanashi, Japan; 3 Fujiyoshida Showa Clinic, Kojinshowa-kai, Kamiyoshida-higashi 1-10-1, Fujiyoshida, Yamanashi 403-0032 Japan

**Keywords:** Hemodialysis, Regeneration, Medication-related osteonecrosis of the jaw, Sequestrectomy

## Abstract

**Background:**

Bisphosphonates are frequently used for osteoporosis. Medication-related osteonecrosis of the jaw, a complication of bone-modifying agents, including bisphosphonates or angiogenic inhibitors, can be challenging to treat in elderly patients with numerous preexisting conditions. Achieving good treatment outcomes is especially difficult in patients with pathological fractures accompanied with extraoral fistulae.

**Case presentation:**

We report an unusual case of prominent bone regeneration following palliative surgical treatment in a 72-year-old Japanese female patient undergoing hemodialysis. She previously had severe osteoporosis due to renal osteodystrophy and was receiving antiresorptive intravenous bisphosphonate. Computed tomography revealed a discontinuous left lower mandibular margin with a pathologic fracture and extensive, morphologically irregular sequestrum formation (80 × 35 × 20 mm). The patient was diagnosed with stage III medication-related osteonecrosis of the jaw and pathologic mandibular fracture. Immediately before the surgery, the anticoagulant used for dialysis was changed from heparin to nafamostat mesylate to reduce the risk of intraoperative bleeding. Sequestrectomy was performed under general anesthesia. Postoperative infection was not observed, the intraoral and submandibular fistula disappeared, and, surprisingly, prominent spontaneous bone regeneration was observed postoperatively at 6 months. Despite the severe systemic condition of the patient, the conservative surgical approach with sequestrectomy has yielded desirable results for more than 6 years since the surgery.

**Conclusions:**

This rare report of spontaneous bone regeneration in a patient of advanced age and poor general condition is the oldest case of mandibular regeneration ever reported.

**Supplementary Information:**

The online version contains supplementary material available at https://doi.org/10.1186/s13256-021-03206-5.

## Background

Bisphosphonates are used to manage metastatic bone disease arising from breast cancer, multiple myeloma, and prostate cancer; in patients with Paget’s disease of bone; and in the treatment of osteoporosis. Medication-related osteonecrosis of the jaw (MRONJ) is defined as exposed bone that can be probed through an intraoral or extraoral fistula in the maxillofacial region, does not heal within 8 weeks, and occurs in a patient with no history of head and neck radiation who has received a bone-modifying agent, including bisphosphonates or an angiogenic inhibitor agent [1, 2]. A long-term complication of these drugs is bisphosphonate-related osteonecrosis of the jaws, first recognized in 2003 [3]. Although most MRONJ cases have been reported in association with intravenously administered bisphosphonates, reports of MRONJ with orally administered bisphosphonates are increasing. Wotton *et al.* reported that oral bisphosphonates were associated with a sixfold increase in the risk of hospital admission with MRONJ in postmenopausal women [4].

The American Association of Oral and Maxillofacial Surgeons (AAOMS) recommends a combination of antibacterial mouth rinse, antibiotic therapy, and analgesics and/or surgical debridement/resection for stage III MRONJ patients (exposed/necrotic bone with pain, infection, and/or pathologic fracture, extraoral fistula, or osteolysis extending to the inferior border) [2]. Others recommend that patients with symptomatic pathologic mandibular fractures undergo segment resection and immediate reconstruction with a reconstruction plate or microsurgical autogenous bone reconstruction [5], but this is controversial because of the possibility of reconstruction failure and the risk of further worsening the overall condition.

Here, we present a rare case of a successful outcome of relatively conservative treatment of a stage III MRONJ patient with a pathologic fracture who was undergoing hemodialysis (HD). Moreover, a comprehensive literature review was performed.

## Case presentation

A 72-year-old Japanese woman was referred to our department She complained of a purulent discharge from a left submandibular fistula and severe pain with hypoesthesia of the left submental region. The patient’s masticatory function was significantly impaired, and she had trouble sleeping because of pain. A panoramic radiograph taken at the first visit showed a fracture of the left inferior mandibular border with minimal deviation. Computed tomography (CT) showed extensive, morphologically irregular sequestrum formation (80 × 35 × 20 mm) in the left mandibular body.

Following initial diagnosis of diabetic nephropathy 13 years earlier, the patient’s disease had already advanced to the point where she needed insulin therapy. Eight years before the referral, diabetic nephropathy progressed further, acute congestive heart failure developed, and emergency dialysis was simultaneously introduced. For the last 8 years, the patient had been undergoing HD every Monday, Wednesday, and Friday morning at 9:00 am for 4 hours owing to kidney failure caused by advanced diabetic nephropathy. Thus, 5 µg of darbepoetin alfa was administered weekly immediately after dialysis to improve renal anemia and maintain hemoglobin level at 10–12 g/dL. To control mineral metabolism and elevated parathyroid hormone (PTH) levels associated with dialysis, the patient was medically managed with active vitamin D3 and sedimented calcium tablets. Then, 7 years before the referral, she underwent a two-branch percutaneous coronary intervention for exertional angina pectoris, and 5 years before the referral, the patient underwent extraction of all lower left molars for periodontitis at a dental clinic, after which the mucosa was completely covered and healed. Due to renal osteodystrophy-induced osteoporosis, she had been receiving monthly antiresorptive intravenous therapy with 1 mg ibandronate sodium hydrate for the last 3 years. Two years before the referral, the patient developed fractures of the second, third, and fourth metatarsals on the left side, which revealed that she had severe chronic kidney disease–mineral and bone disorder (CKD-MBD) [6] with a young adult mean (YAM) value of 45%. Six months before the referral, the patient began experiencing pain in the left mandibular body and paralysis of the mental region, and she visited her primary care dentist for diagnosis and treatment. CT scan revealed normal findings in the left mandibular body, with no tooth or foreign substances as a source of infection, and no jawbone exposure or bone resorption. Although the cause could not be identified, continued observation revealed bone exposure, and the patient’s condition deteriorated. She was then referred to a public municipal hospital where stage III MRONJ was diagnosed, based on the AAOMS guidelines [2]. She was treated in the hospital with regular flushing and antibiotic therapy; however, the local infection gradually worsened, and the patient was referred to our department.

**Fig. 1:**
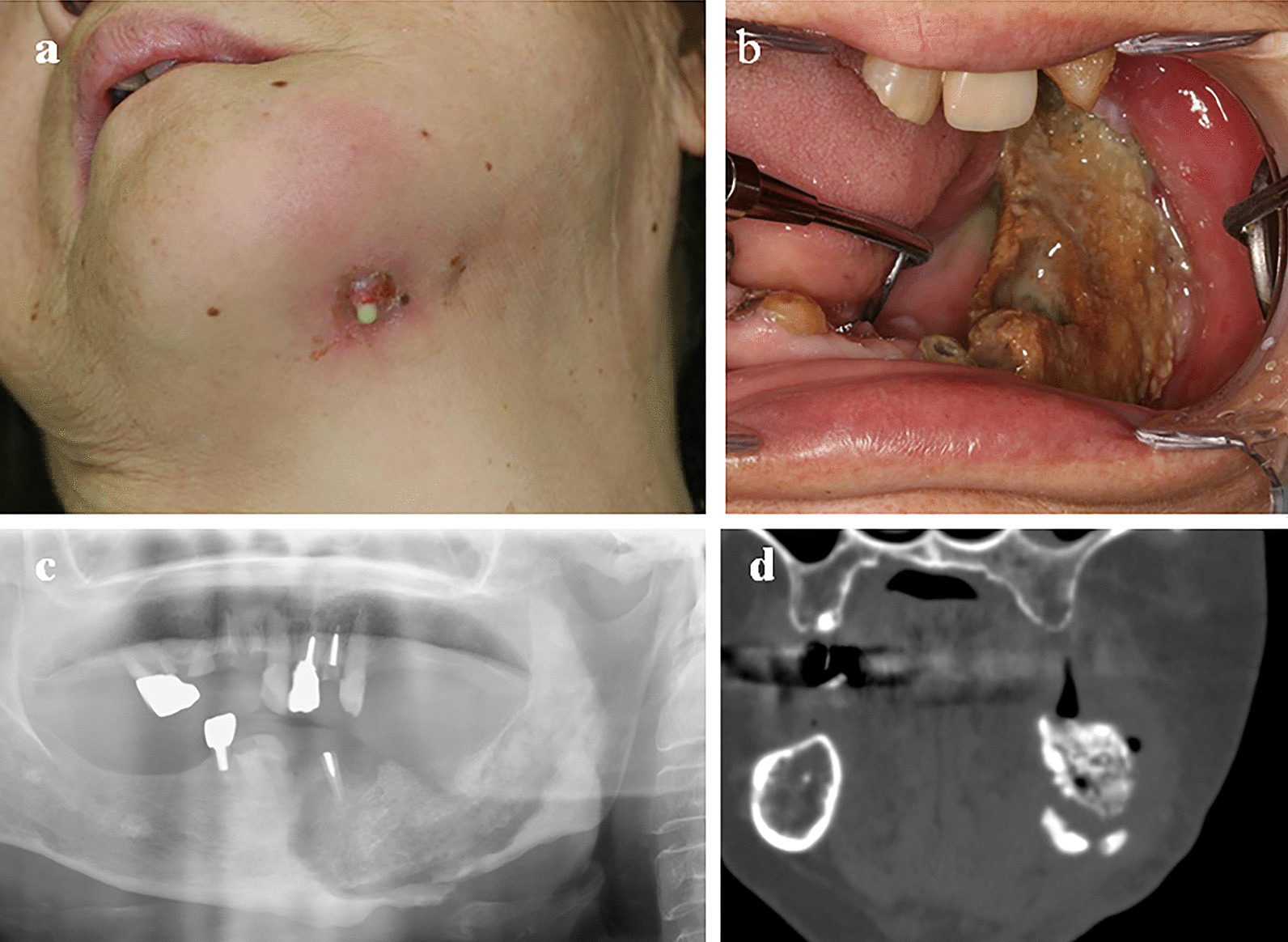
Preoperative appearance and imaging results of our patient. **a** Lower facial appearance showing a chronic inflammatory reaction with a pustular discharge. **b** Intraoral view showing necrotic bone and mucosa inflammation on the left half of the mandible. **c** Panoramic radiograph showing a large sequestrum with a fracture of the left mandible. **d** Computed tomography scan showing sequestrum and fracture in the left mandibular plane

**Fig. 2:**
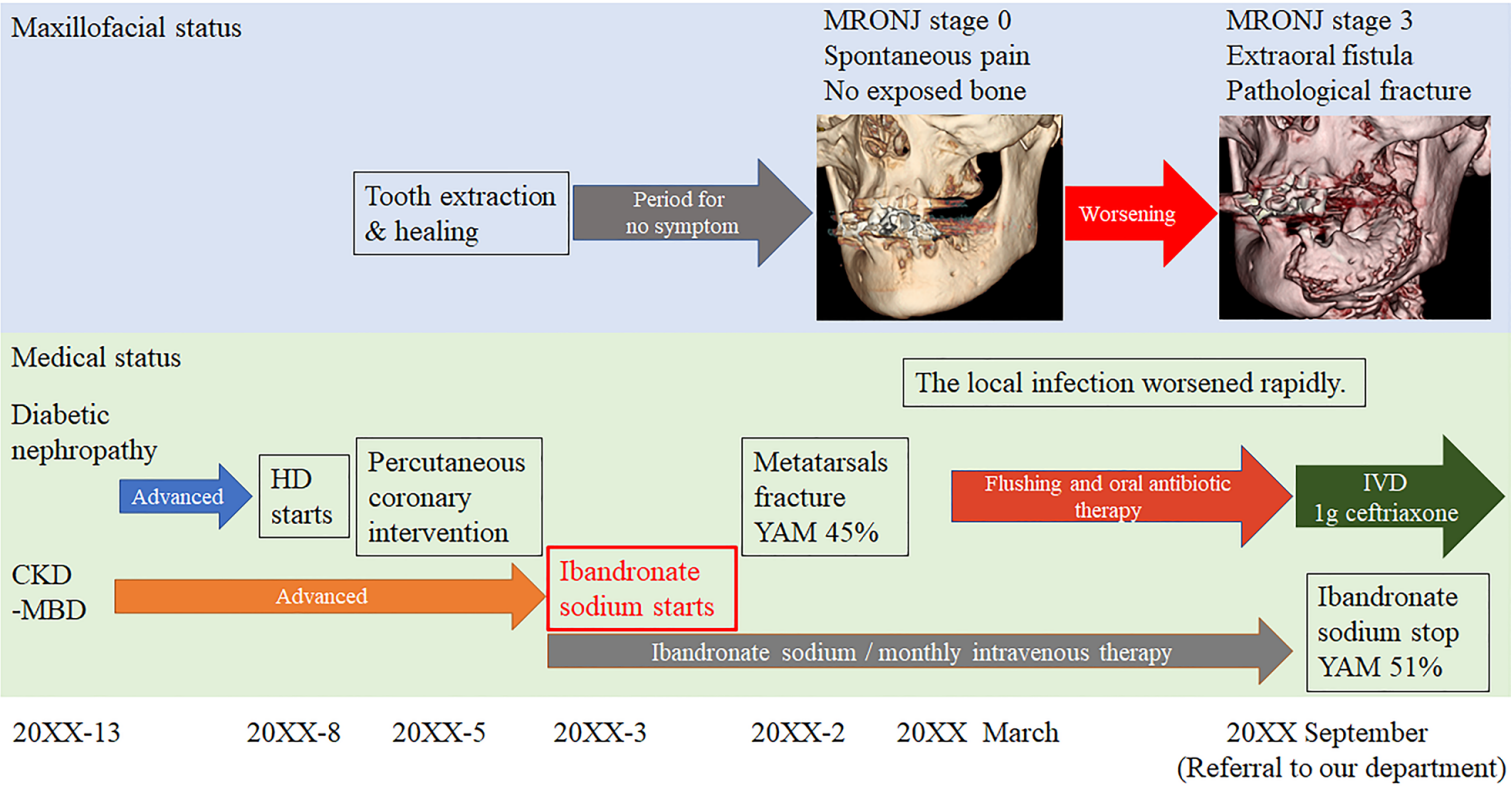
The patient’s clinical course leading up to surgery for sequestrectomy. *CKD-BMD* chronic kidney disease–mineral and bone disorder, *IVD* intravenous drip, *HD* hemodialysis, *MRONJ* medication-related osteonecrosis of the jaw, *YAM* young adult mean. “20XX” indicates the year when the patient was first referred to our department

Preoperative blood sampling showed elevated levels of C-reactive protein (CRP), suggesting a systemic as well as a local inflammatory response (Table 1). The abscess was drained, and daily intravenous antibiotic treatment of 1 g ceftriaxone sodium hydrate commenced. Additionally, ibandronate sodium hydrate administration was discontinued after consulting with the doctor responsible for the patient’s dialysis given that the YAM value had recovered to 51%. A panoramic radiograph revealed a displaced pathologic fracture on the left side of the atrophic mandibular body, corresponding to stage III MRONJ (Fig. 1). Although the acute inflammation disappeared with anti-inflammatory treatment, the cutaneous fistula worsened, and the deviation of the pathologic fracture of the mandible increased. Figure 2 shows the clinical course from the onset of diabetic nephropathy leading up to the surgery for sequestrectomy. Consequently, the patient underwent decortication and fistula closure under general anesthesia 2 months after the referral. Immediately before the surgery, the anticoagulant used for dialysis was changed from heparin to nafamostat mesylate to reduce the risk of intraoperative bleeding. Because she also had cardiac disease, she was managed in the intensive care unit during the perioperative period. Intravenous injection of 1 g cefazolin sodium was administered at least 30 minutes prior to the surgery to prevent surgical-site infection. Oral hygiene practices were performed daily by an oral surgeon (KY) during the hospitalization, and cefcapene pivoxil hydrochloride hydrate was orally administered at a dose of 100 mg once daily for 6 weeks after the surgery. The antibiotic dose was reduced to one-third of the usual dose because the patient was under dialysis management. Antibiotics were administered for a relatively long period of 6 weeks until complete mucosal closure, and the route of wound infection was completely eliminated. After confirming that swallowing function was intact using the water swallowing test, the nasogastric tube was removed on the second postoperative day, and a soft dialysis diet was initiated. The sequestrectomy and fistula closure were successful, and no abnormal bleeding or postoperative infection was observed. Intraoperatively, the periosteum was left intact, and the sequestrum was dissected from the cortical surface (subperiosteal resection). The general perioperative condition of the patient was well maintained under HD management, and there were no cardiac events.

**Table 1:** Comparison of hematologic and biochemical parameters before surgery

Variable	Before surgery	2 years after surgery	Reference, range
Albumin (mg/dL)	3.1	3.4	4.1–5.1
WBC count (/µL)	8130	6440	3500–8600
Neutrophil (/µL, %)*	6500, 79.9%	4600, 71.4%	1470–6364, 42.0–74.0%
Hb (g/dL)	9.9	10.4	11.2–14.8
Hematocrit (%)	27.8	33.8	34.3–45.2
ALP (U/L)	134	116	105–320
CRP (mg/dL)	3.08	0.20	0–0.30
Serum-corrected calcium (mg/dL)	10.3	9.5	8.4–10.0
Serum phosphorus (mg/dL)	5.5	6.0	3.5–6.0
Intact PTH (pg/mL)	68	115	60–240
YAM (%)	50.4	50.6	70 ≦
Urine volume (mL)	300	100	–

*WBC* white blood cell, *Hb* hemoglobin, *ALP* alkaline phosphatase, *CRP* C-reactive protein, *PTH* parathyroid hormone, *YAM* young adult mean

*Neutrophils are expressed as the absolute number/µL and as the % of WBCs.

Histopathology revealed osteonecrosis and the presence of *Staphylococcus* species and Gram-negative bacilli that were identified as facultative anaerobes, and there was no evidence of tumor disease. During the first 3 months after surgery, the wound was regularly cleaned, and a soft diet was followed to avoid force loading.

Surprisingly, the 6-month follow-up CT scan and panoramic radiograph showed spontaneous bone regeneration. The fracture was replaced by new bone, bone healing in the periosteum was observed, and the patient was able to eat with a denture. Furthermore, the Semmes–Weinstein monofilament (SAKAI Medical Co., Ltd., Tokyo, Japan) examination performed 6 months after surgery revealed that the patient could identify the lowest target force (0.008 g) as a sensation and that thigmesthesia had recovered to normal, with no difference between the left and right sides of the submental region.

**Fig. 3:**
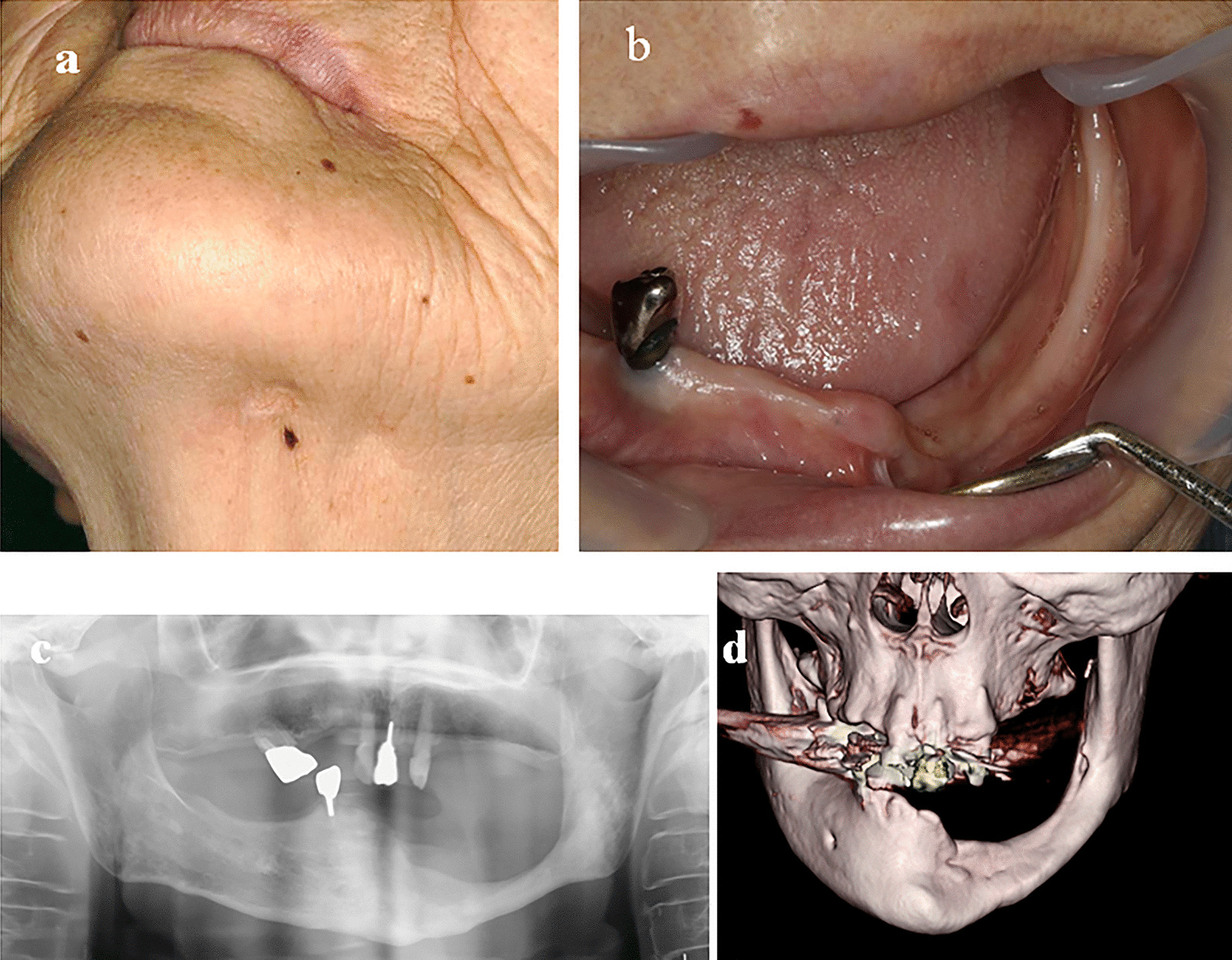
Appearance and imaging results of our patient 3 years after surgery. **a** Lower facial appearance showing with no inflammatory reaction or fistula. **b** Intraoral view showing no exposed bone. **c** Panoramic radiography showing regenerated bone of continuous thickness in the left mandible. **d** Three-dimensional computed tomography showing the preserved mandibular foramen and thick, continuous bone

Blood sampling after the surgery showed CRP within normal limits and an improvement in hematologic and biochemical values. The patient’s oral condition remained good, with no findings of infection to date (Fig. 3, 3 years after the referral; Additional file 1: Fig. S1 shows the most recent X-ray imaging findings of spontaneous bone regeneration 6 years after the referral).

### Literature review

A literature search was conducted in PubMed according to the following method reported by Matsuda *et al.*: [7] (spontaneous [Title]) AND (regeneration [MeSH Terms]) AND (mandible [MeSH Terms]) AND (“2003” to “Present” [Data-Publication]).

Eleven papers were identified by our literature search [7–17], including 26 patients (15 males and 11 females; mean age 23.2 ± 15.7 years). Ameloblastoma was the most commonly treated disease (12 cases), followed by ossifying fibroma (6 cases). The oldest patient, aged 70 years, was a case of MRONJ [7]. Immediate reconstruction was reported as performed in seven cases, most of which were segmental mandibulectomy with preservation of the periosteum to achieve spontaneous bone recovery.

## Discussion

Based on our literature review, to date, this is the oldest patient with stage III MRONJ who presented with mandibular spontaneous regeneration. Furthermore, she had a cardiovascular risk and required strict management, including weight gain/loss monitoring and antithrombotic therapy during HD [18]. Differentiating between odontogenic osteomyelitis and MRONJ is necessary. Accordingly, the current patient was diagnosed with MRONJ because all teeth in the same area had been extracted and the site had completely healed 2 years before the initiation of treatment with ibandronate sodium hydrate. The sequestrum was carefully removed to maintain an intact periosteum. Unexpectedly, bone healing was observed, and thigmesthesia was recovered to normal at the 6-month checkup.

Whether a normal or reduced dose of bisphosphonates is optimal during dialysis management remains controversial. Miller *et al.* reported that intravenous bisphosphonate formulations in patients with HD require either a reduced dose or a slower infusion rate, given that bisphosphonate administration with poor renal clearance causes higher bone retention [19]. However, Bergner *et al.* reported that a monthly dose of 1 mg ibandronic acid did not increase plasma levels in patients with stage 5 CKD undergoing hemodialysis three times per week because it is efficiently removed by dialysis [20]. Considering that the patient developed ARONJ after 3 years of treatment at the standard dose, a dose reduction may have been necessary. Nonetheless, more data are needed to determine the risks and benefits of bisphosphonates in patients with stage 5 CKD.

Previous studies have demonstrated that the exposed sequestrum was significantly contaminated with *Staphylococcus* and Gram-negative rods and that the oral cavity was also contaminated [21]. Postoperative bone regeneration, which has been observed after surgical resection for the treatment of tumors [12, 22], osteomyelitis [23], and MRONJ [24], is attributed to periosteum maintenance [25]. In fact, studies have reported spontaneous bone regeneration in the jaw, malleolus, and tibia after injuries [26]. In the current case, considering the timing of bone remodeling, treatment with ibandronate sodium hydrate was discontinued for approximately 3 months before surgery [27]. However, evidence on the association between discontinuation of bisphosphonate treatment and better clinical outcomes in MRONJ has been controversial [28, 29].

The periosteum is a specialized connective tissue composed of a thin and tough fibrous membrane that is attached to the surfaces of bones. It consists of an outer surface layer containing fibroblasts, blood vessels, and Sharpey’s fibers and an internal cambium layer containing capillaries and osteoblasts. The inner membrane plays a significant role in bone regeneration and remodeling. Periosteum preservation, patients of a young age, and the presence of a low-grade infection, such as chronic osteomyelitis, are factors that stimulate periosteal bone regeneration [30]. In our case, the patient was of older age, and infection was not observed during the postoperative period, suggesting that preservation of the periosteum may be the most crucial factor in activating bone regeneration. The periosteal surface is the most important region for bone healing, and the preservation of the periosteum may have resulted in the dramatic bone regeneration observed in our case. Moreover, there were some areas with only periosteum and no bone, and solid bone regeneration was observed in these areas following surgery. Therefore, we believe that preservation of an intact periosteum is a critical stimulation factor in spontaneous bone regeneration, even in patients of an older age with a poor general medical condition. Spontaneously formed bone is usually radiographically observed at 3 months after surgery [15]. However, bone regeneration in our case was only observed radiographically at 6 months after the surgery. This delay in regeneration may have been due to her advanced age and poor general condition, and there were no adverse clinical effects.

## Conclusions

In conclusion, despite the severe systemic condition of this patient, the conservative surgical approach of sequestrectomy has yielded desirable results for more than 6 years to date. Additionally, this patient is the oldest case of mandibular regeneration ever reported.

## Supplementary Information

**Additional file 1: Fig. S1.** Imaging findings showing the most recent state of the patient. Panoramic radiograph showing continuous thickness of regenerated bone in the left mandible, which has been maintained to date.

## Data Availability

All data generated and/or analyzed during this study are included in this published article.

## Abbreviations

AAOMS: American Association of Oral and Maxillofacial Surgeons
CRP: C-reactive protein
CT: Computed tomography
HD: Hemodialysis
MRONJ: Medication-related osteonecrosis of the jaw
