# Supplementary figures and images for: An unusual case of bone regeneration of a necrotic mandible with pathologic fracture in an elderly hemodialysis patient with medication-related osteonecrosis of the jaw: a case report and review of the literature
Source: J Med Case Rep. 2021 Dec 23;15:608. doi: 10.1186/s13256-021-03206-5 (PMC8697441; doi:10.1186/s13256-021-03206-5)

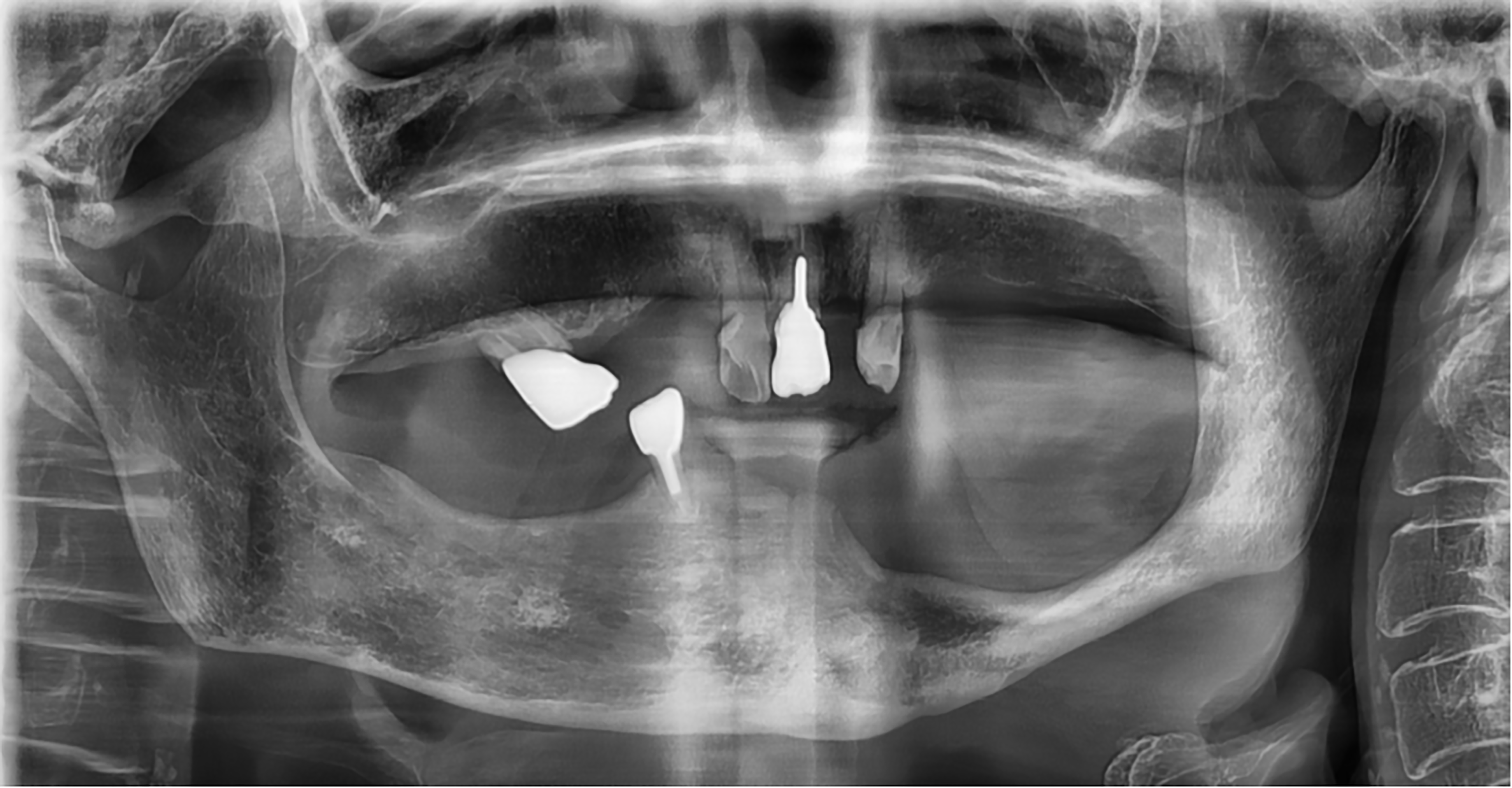

Supplement: Supplementary file 1 — Additional file 1: Fig. S1. Imaging findings showing the most recent state of the patient. Panoramic radiograph showing continuous thickness of regenerated bone in the left mandible, which has been maintained to date. [file 13256_2021_3206_MOESM1_ESM.tif]
